# The Interplay Between Sugar and Yeast Infections: Do Diabetics Have a Greater Predisposition to Develop Oral and Vulvovaginal Candidiasis?

**DOI:** 10.7759/cureus.13407

**Published:** 2021-02-18

**Authors:** Lubna Mohammed, Gaurav Jha, Iana Malasevskaia, Harshit K Goud, Aiman Hassan

**Affiliations:** 1 Internal Medicine, California Institute of Behavioral Neurosciences & Psychology, Fairfield, USA; 2 Cardiology, Narayana Hrudayalaya, Bangalore, IND; 3 Neurology/Stroke Medicine, Queen's Hospital, London, GBR; 4 Obstetrics and Gynaecology, California Institute of Behavioral Neurosciences & Psychology, Fairfield, USA; 5 Family Medicine, California Institute of Behavioral Neurosciences & Psychology, Fairfield, USA

**Keywords:** type 1 and type 2 diabetes mellitus, vulvovaginal candidiasis, oral candidiasis, candida, candidal infection, female diabetics, unmanaged diabetes, pre-diabetes, immunocompromised hosts, chronic disease care

## Abstract

Diabetes mellitus (DM) is one of the most common chronic diseases impacting individuals of both developing and developed nations. DM patients have a weaker immune system in comparison to healthy subjects, rendering them more prone to develop infections. Even the typical gut microflora can become pathogenic in such immunocompromised conditions. Microorganisms belonging to Candida species are capable of causing infections in DM subjects. A comprehensive review of the literature was undertaken. The PubMed database was searched using well-defined search terms. Predefined inclusion and exclusion criteria were applied to classify relevant manuscripts. The results of the review show that DM patients have an increased susceptibility to Candida sp. This paper will summarize the previously conducted research discussing the relationship between DM and candidiasis, features specific to Candida species that make it pathogenic, and compare oral and vulvovaginal candidiasis (VVC) morbidity in diabetics versus healthy subjects.

## Introduction and background

**Figure 1:**
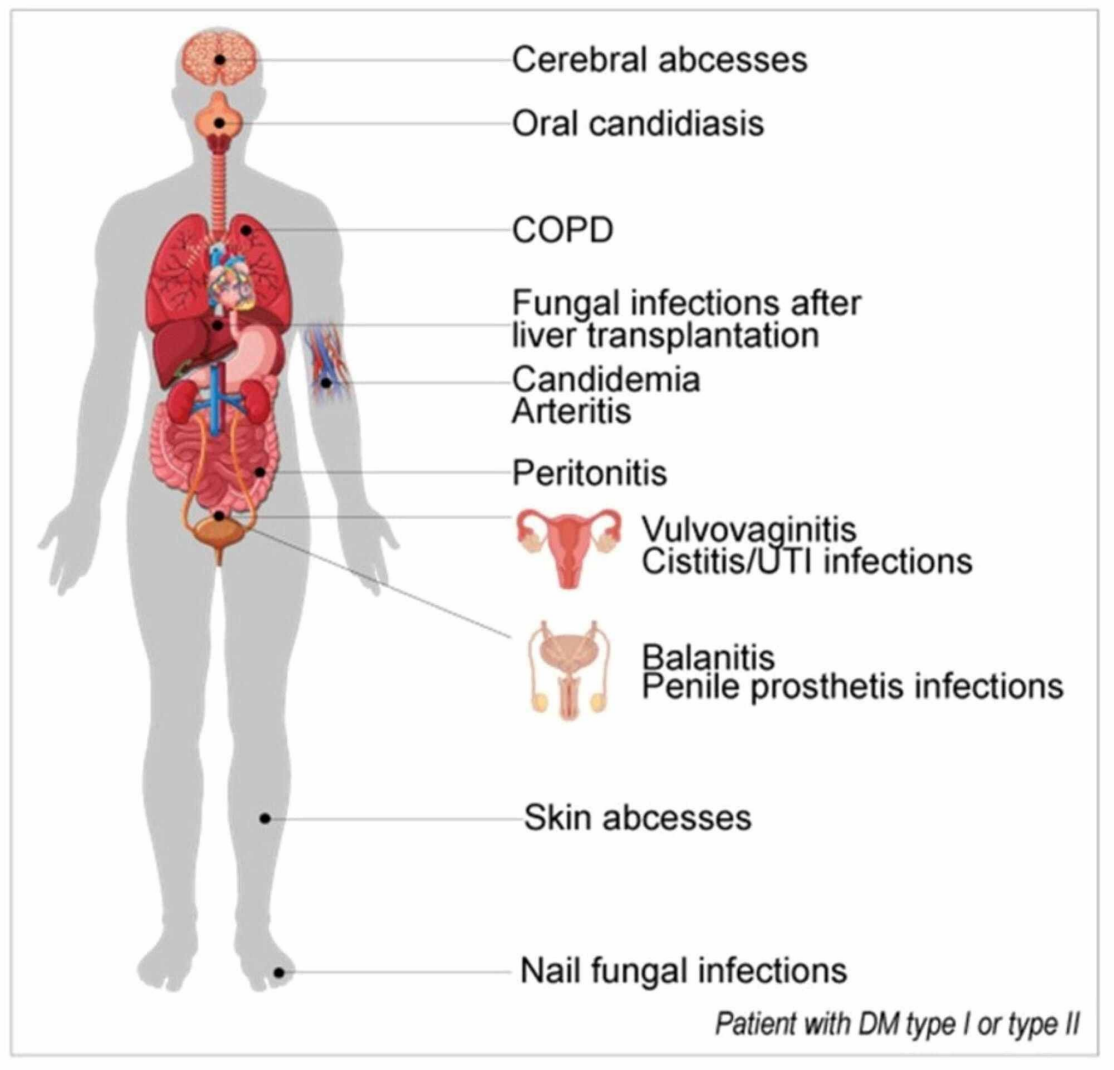
List of diseases that Candida species can cause with greater incidence in diabetics.

Diabetes mellitus (DM) is one of the most prevalent endocrine disorders impacting millions of individuals worldwide, signified by the presence of long-standing hyperglycemia [1]. Type 1 DM is characterized by the complete deficiency of insulin because of the destruction of the pancreatic beta cells, whereas type 2 DM is caused due to insulin resistance, which may eventually lead to a hyperglycemic state [2]. The presence of constantly high glucose levels in the blood favors an environment suitable for damage to the blood vessels of all sizes, resulting in microangiopathy and macroangiopathy (atherosclerosis) [1]. There is a significant impact in the form of neuropathies and nerve disintegration of both the central and autonomous nervous systems because of the insufficient blood supply to the nerves and the hyperglycemic environment [1]. DM can also lead to a compromise in cellular immunity [3]. A series of inflammatory reactions occur because of insulin resistance, as insulin signaling stoppage causes aggravation to the pre-existing inflammatory milieu due to metabolic disturbances in DM [3]. In accordance with the American Diabetes Association, diabetic patients suffer from a major problem of a weak immune system that hampers their capability to fight intrusive microorganisms, rendering them a higher predisposition to infections [3]. The recovery time from infections or injuries in individuals with DM is significantly prolonged in comparison to the healthy population [1]. Figure 1 lists the diseases that Candida species can cause with greater incidences in diabetics.

Type 2 DM is being referred to as an emerging worldwide pandemic of the 21st century, as it was earlier prevalent in only powerful developed nations but now has spread worldwide, affecting people of developing countries as well [6]. In 1964, an estimation was done that 30 million individuals had DM. However, the counts rose to 171 million individuals in a span of lesser than 40 years [7]. The universal prevalence of DM is shown to have a drastic rise, especially in the recent past. In 2015, an estimation was made that 415 million patients within the age group of 20-79 years were found to be diabetic, accounting for an 8.8% prevalence worldwide. It is being assumed that the counts might increase up to 642 million persons by the year 2040, making the prevalence rate 10.4% worldwide [7]. The younger population group affected by fungemia were reported to have greater rampancy of liver diseases, acquired immunodeficiency syndrome (AIDS), and organ transplants. The older individuals were reported to have a significant existence of DM and cardiopulmonary diseases [8]. The link between DM and candidiasis has been a topic of in-depth study because of the greater predisposition of diabetic individuals to develop candidiasis in contrast to the healthy population [3]. This review article will summarize the reasons for higher incidences of candidiasis in DM patients.

Search strategy

In-depth research was performed by utilizing the keywords in Table 1 to collect the studies that analyze and assess the relationship between the prevalence of Candidiasis in diabetic patients using PubMed as the main database. Apart from the study's primary aim, the causes for the pathogenicity of Candida sp. and the reasons for affecting DM patients are also discussed in this study. The inclusion criteria consisted of articles published within the last 21 years (2000 - January 2021). The chosen articles had to be available in the English language on the primary database. There was no restriction on the study type, and all kinds of articles like traditional reviews, systematic reviews, case-control studies, cohort studies, and clinical trials were scanned. There were no demographic restrictions during the review process. Age and ethnicity were also not used for refining the study. Exclusion criteria comprised incomplete articles, publications whose abstracts were unavailable, and articles published before 2000. The keywords and their search results for procuring the data are summarized in Table 1.

**Table 1:** Keywords and their search results

Keywords	Database	Results
Diabetes mellitus	PubMed	359,083 results
Candida	PubMed	46,691 results
Candidiasis	PubMed	20,325 results
Oral candidiasis	PubMed	4,811 results
Vulvovaginal candidiasis	PubMed	1,868 results
Candidiasis and diabetes mellitus	PubMed	606 results
Oral candidiasis in diabetics	PubMed	289 results
Vulvovaginal candidiasis in diabetics	PubMed	96 results

## Review

Discussion

This section will discuss the diagnostic criteria of DM, the pathogenesis of Candida sp. infections in DM subjects, oral candidiasis studies, and VVC studies that demonstrate a greater predisposition of these infections in people with DM when compared to euglycemic subjects.

DM and Candidiasis

DM is a very prevalent endocrine disease that renders the individual very prone to getting infections because of immune damage. Multiple factors are involved like a drop in T-lymphocyte counts, reduced action of neutrophils, rise in programmed cell deaths of leukocytes, and reduction in the release of cytokines [9]. All of these factors get aggravated because of the hyperglycemic environment in diabetic patients [9]. Recurring candida infections are a prominent sign of DM that sometimes facilitates the identification of an individual's pre-diabetic condition [10]. An individual is suspected of having a pre-diabetic condition or impaired glucose tolerance when their fasting plasma glucose is within the range of 100-125 mg/dL [11]. The diagnostic criteria of DM are summarized in Table 2 below.

**Table 2:** Diagnostic criteria of DM DM-Diabetes Mellitus

The diagnosis of DM by the World Health Organization (WHO) [10]	The diagnostic criteria of DM by the American Diabetes Association (ADA) [10]
-Fasting plasma glucose level ≥ 7 mmol/L or 126 mg/dL	-A patient with typical symptoms of hyperglycemia like polyuria, polydipsia, polyphagia, weight loss, or hyperglycemic crisis showing random plasma glucose levels to be ≥ 200 mg/dL or 11.1 mmol/L
-Two hour plasma glucose level ≥ 200 mg/dL or 11.1 mmol/L	-National Glycohemoglobin Control and Complications Trial (DCCT) certified laboratory method, which detects the hemoglobin A1c (hbA1c) levels to be ≥ 6.5% or 48 mmol/mol
	-Fasting plasma glucose level ≥ 7 mmol/L or 126 mg/dL
	-Two-hour plasma glucose level ≥ 200 mg/dL or 11.1 mmol/L

**Figure 2:**
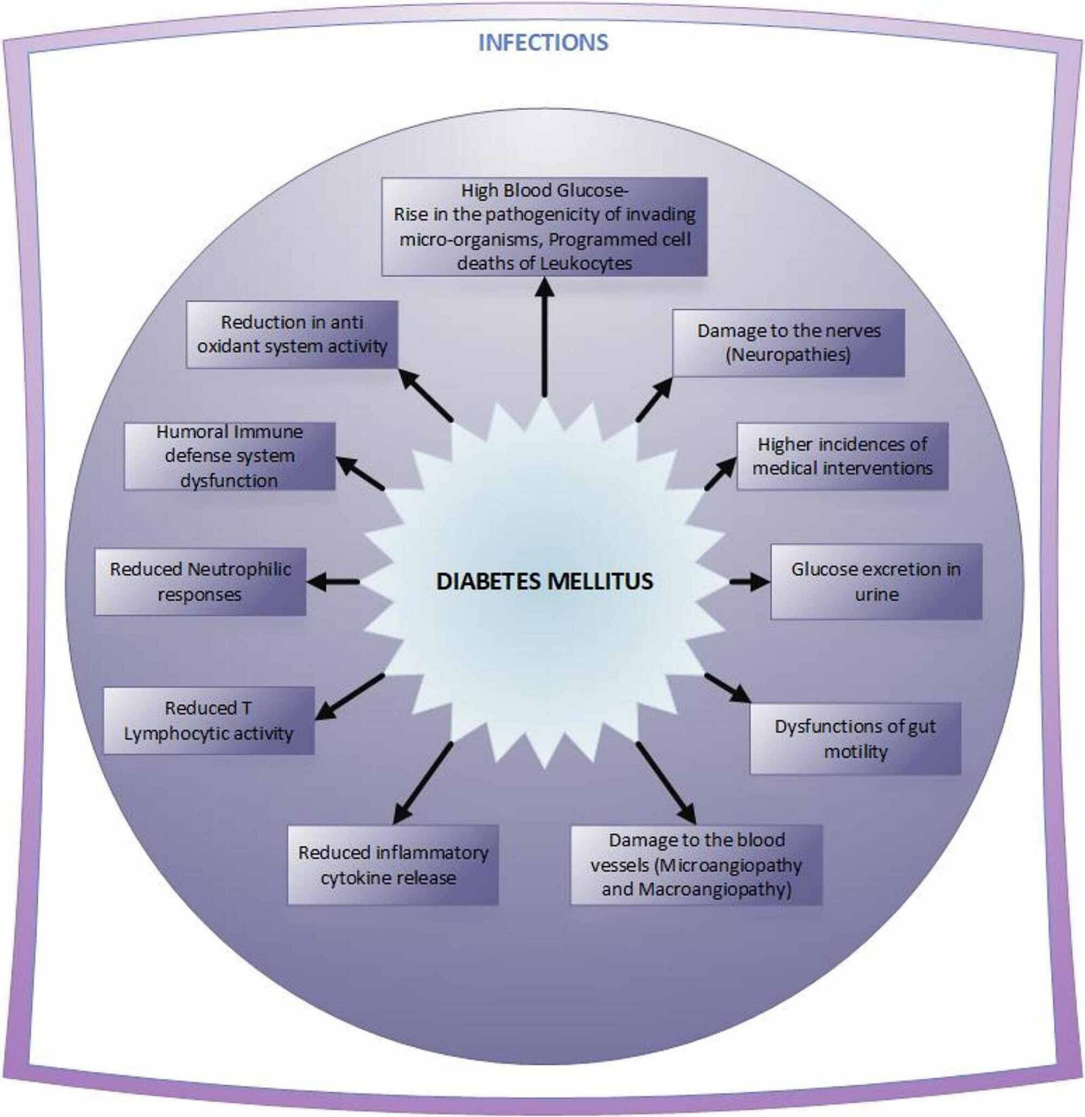
Reasons for the predisposition of diabetics to infections

DM patients have a higher risk of developing opportunistic infections and multiple organ damage, which deteriorates their capability to fight off invading pathogens [3]. Figure 2 depicts the reasons for the greater susceptibility of diabetics to infections [11].

Candida species have a broad distribution pattern and are commonly seen in human beings, domestic and wild animals, and diverse environments such as hospitals [12]. Candida species are a constitution of typical human microflora and possess the colonizing abilities of the mucosal surfaces such as the oral cavity, gastrointestinal tract, respiratory tract, and genitourinary tract [12]. However, Candida species are capable of either existing as a commensal or transforming symptomless colonies into infective [12]. The pathogenic capabilities of Candida species and their colonization factors are dependent on host-related immune factors due to an intricate homeostatic relationship linking the fungi and the host's current immune condition, which is the key determinant of either commensalism or parasitism [13]. There are several recognized factors that have a significant impact on homeostasis and make Candida colonization more susceptible. These factors aid its transition from the commensal to the pathogen. Some of these factors include the adhesive nature of Candida species to the host's epithelial cell surfaces, raised glucose levels in the saliva, reduction in the flow rates of the saliva, degradation of the microvasculature, and weakened immune response of neutrophils for candidacidal activities [3].

Hyperglycemia, the release of numerous degenerative nature enzymes, or even an immune-suppressed host result in the above-stated conditions becoming strikingly critical for the patient [3]. The predisposition for creating a suitable environment for Candida multiplication includes various factors such as reduced gut secretions, dietary causes, alteration of the gastrointestinal microflora, immuno-compromised condition and co-existing morbidities, continued usage of antibiotics, or other drugs, altered liver functionality, deficiency of necessary nutrients [14].

Pathogenic features of Candida species

Candidalysin

The yeast Candida albicans can produce a toxin that causes cytolysis called "candidalysin" [15]. The discovery of candidalysin toxin has been fairly recent. Significant in-depth research is the dire need of the hour in this regard, as Candida species are regular commensals of the human microbiome, and they possess remarkable abilities to initiate the series of events linked with disease and can cause significant consequences [16]. The hyphae of Candida albicans are responsible for secreting candidalysin, which plays a vital role in oral or vaginal mucosal infections by activating epithelial cells to initiate an innate response downstream related to protection or causing immunopathology in those infections [17]. It is an immune-modulatory molecule that is recognized as one of the critical factors for activating host cell responses, recruiting neutrophils, and causing direct damage to the host cells [15]. This toxin also causes the influx of calcium ions and helps in releasing lactate dehydrogenase (LDH) from the oral epithelial cells that thereby cause membrane destabilization and cell destruction [15]. During disseminated systemic yeast infections, candidalysin is supposed to play a vital role in recruiting neutrophils and aiding yeast virulence [17].

Secreted Aspartyl Proteinases (Sap Proteins)

Aspartyl proteases exist in several organisms, and among the pathogenic species, they are regarded as one of the major virulence factors [18]. Diversified virulence factors aid the pathogenicity of fungal Candida species, but secreted proteolytic enzymes are being studied for their disease establishing and progressing abilities [18]. The secretion of proteolytic enzymes like Sap proteins helps in the pathogenicity of Candida species by expediting the infection by helping in the digestion of molecules for acquiring nutrients [19]. They aid in pathogenicity and invasiveness by confiscating immune responses. They aid in concealing from host immune cells by breaking down their anti-microbial response cells and molecules [19]. Digestion and distortion of the host's cell membranes to aid yeast's adhesiveness and tissue invasiveness is also a feature of Sap proteins [19]. They have the ability to alter the host complement cascade and can also cause macrophages to release cytokines or induce chemotaxis [18].

Biofilm Formation

Biofilms are colonies of microflora lodged in an extracellular matrix that offer considerable resistance to any anti-microbial therapy and cause a spike in the host's immune response [3]. The distinct features of a biofilm comprise irreversible attachment to a surface or common boundary between adjacent regions, extracellular polymeric matrix (PEM) embedding abilities also involving the abiotic and non-cellular elements produced by themselves, special physical characteristics like altering the growth rates [20]. Biofilm microflora can transcribe genes that the other microorganisms are incapable of [20]. So, the current definition of a biofilm can be summarized as "a microbially derived sessile community characterized by cells that are irreversibly attached to a substratum or interface or each other, are embedded in a matrix of extracellular polymeric substances that they have produced, and exhibit an altered phenotype with respect to growth rate and gene transcription" [20].

There are four sequential steps involved in the production of Candida species biofilms, which are summarized in Table 3 [21].

**Table 3:** Steps involved in the production of Candida biofilm Polymeric matrix-PEM

Biofilm formation [21]-
Adherence- In the first three hours, the initiation of the yeast's adhesion in suspension and other planktonic cells to the surface takes place.
Intermediate phase- In 11-14 hours, the formation of biofilm occurs.
Maturation phase- In 20-48 hours, complete penetration of all the layers of cells attached to the surface by the PEM in a three-dimensional pattern.
Dispersion- After a duration of 24 hours, the top-most superficially attached cells decamp from the biofilm and start colonizing the surrounding surface areas.

Therefore, the composition of a fully formed biofilm consists of a dense network of yeast cells, hyphae, or pseudohyphae (variates according to the strain of the Candida species involved) involving PEM along with water channels linking the cells [3].This aids in diffusing nutrients acquired from the environment to reach the lowermost layers by penetrating through the biomass and helps in removing waste materials [21]. Previous research has proved that Candida species that were isolated from DM subjects possess more incredible pathogenic abilities for biofilm production [22]. The hyperglycemic environment supposedly serves as a carbohydrate energy-generating resource that may be a requirement for the Candida species biofilm and matrix production for protecting themselves [3]. Biofilm production aids the yeasts to become more resistant to anti-fungal therapy than infections caused by other microflora [23]. The biofilm communities are a usual occurrence on medical devices, and the reduction of biofilm-related candidiasis is the need of the hour for providing optimum patient care [23].

Oral Candidiasis

Candida species are regular inhabitants of the buccal cavity. However, raised glucose levels in the saliva offer a suitable environment for the flourishment of Candida species, which results in oral candidiasis and dental plaques [24]. DM, especially when uncontrolled, leads to the development of oral candidiasis in around 25% of the patients [24]. The previously conducted studies demonstrated that the saliva of diabetic individuals contained greater Candidal colony-forming units when compared to the saliva of healthy subjects [24-25]. A higher incidence of Candida species is found in the DM patient's oral cavity due to various factors like reduced salivary flow rates, higher concentrations of salivary glucose, diminished host defense system due to reduced neutrophilic activities, and greater adherence of Candida species to buccal epithelial cells [26]. The findings of the studies included in this review regarding the prevalence of oral candidiasis in DM patients are summarized in Table 4.

**Table 4:** Studies summarizing the prevalence of oral candidiasis in DM subjects in comparison to healthy subjects DM-Diabetes Mellitus, n-Numbers

Author	Year	Study population	Findings
Jhungroo C et al., [27]	2019	500; Numbers (n)=250 for DM patients and n=250 for healthy subjects.	Higher incidence of Candida sp. in people with diabetes when compared to non-diabetics.
Anari et al., [28]	2019	302; n=151 for pre-diabetics, n=151 for healthy individuals.	Statistically significant differences were found in the oral cavity pathologies, including Candidiasis of the pre-diabetic individuals compared to the healthy group. Candidiasis was found to be frequently prevalent in the pre-diabetic group.
Bissong et al., [29]	2015	251; n=149 diabetics and n=102 for healthy subjects.	21.5% of the diabetic patients showed Candidiasis along with the increased incidence of other oral diseases, the root cause being identified as hyperglycemia
Kumar et al., [30]	2015	103 DM subjects; n=49 of type 1 DM, n=54 of type 2 DM; 100 control subjects.	83.67% of type 1 DM subjects had Candida prevalence in the buccal cavity. 68.52% prevalence rates in type 2 DM individuals. Only 27% occurrence was found in healthy subjects.
Mohammadi et al., [31]	2016	106 subjects; n=58 for diabetics, n=48 for healthy subjects.	55% of the diabetic patients showed the prevalence of Candida species, and around 50 colonies were isolated. Only 27% of the healthy subjects showed the presence of Candida species in the oral cavity.
Belazi et al., [32]	2005	212 subjects; n=128 for DM, n=84 for healthy subjects.	64% of the diabetic subjects showed the presence of Candida albicans in comparison to only 40% of the healthy subjects.

Vulvovaginal Candidiasis (VVC)

VVC is an emerging health issue worldwide, as it impacts millions of females due to the vaginal mucosa becoming superfluous flourished by yeasts [33]. The majority of the females are hosts to Candida species at certain points in their life. However, they can remain asymptomatic because of vaginal host defenses that keep their multiplication in check [12]. Candida species were identified in around 70% of females over the course of a one-year surveillance duration, and their colonies were isolated from 33.33% of females, but they remained asymptomatic [34]. Out of control blood glucose levels result in various metabolic changes like a rise in glycogen levels. This can cause a remarkable increase in the colonizing and pathogenic abilities of Candida species, as raised glycogen levels cause a fall in the pH of the vagina, making the environment more susceptible to the establishment of a VVC community [26]. During their reproductive age, around 75% of the females get affected with at least one episode of VVC, and roughly 50% of them have two or more incidences [34]. De Leon et al. found that the frequency of Candida colonization was three times higher in type 1 DM in comparison to type 2 DM patients in a study comprising 101 DM subjects [35]. In the same study, the most prevalent colonies were of Candida albicans, isolated from 56% of type 1 DM subjects, whereas Candida glabrata had a 54% prevalence rate of colonies in Type 2 DM patients [35].

The disease spectrum comprises a plethora of signs and symptoms like white discharge, which is thick cottage cheese-like, vulvovaginal itching, pain, redness, burning, edema. Sometimes, eventuating dysuria and dyspareunia can also be seen [33]. The women who were suffering from VVC had higher mean hbA1c levels than subjects without any such infections. Therefore, researchers suggest that routine periodical screening for VVC should be offered to DM patients [3]. Table 5 summarizes the studies that indicate a greater predisposition of diabetics to develop VVC.

**Table 5:** Studies comparing the incidence of VVC in diabetics versus healthy subjects DM-Diabetes Mellitus, VVC-Vulvovaginal Candidiasis, n-Numbers

Author	Year	Study Population	Study Outcomes
Gunther et al., [26]	2014	717 women screened for Candida species; Diabetic group Numbers (n); n= 48, Control group n=669.	Type 2 DM patients in Brazil exhibited noteworthy greater colonization, symptoms, and recurrent infections when compared to the healthy group. 18.8% of diabetics showed vaginal Candida species whereas only 11.8% rates in normal subjects.
Goswami et al., [30]	2000	166; n=78 Diabetics, n=88 non-diabetics.	46% (36/78) of DM patients showed Candida species and only 23% (21/88) normal subjects demonstrated Candida species.
Kendirci et al., [36]	2004	57; n=35 Type 1 DM, n=22 healthy subjects.	52.5% (32/61) of the Type 1 DM samples that were collected had shown the presence of Candida species whereas only 18.2% (5/22) of the healthy subjects showed its presence.
Donders et al., [37]	2002	94 women; n=62 women had ≥ three episodes of Candidiasis and positive Candida findings in microscopy, n=32 for healthy Candida negative controls.	36% of patients with recurrent VVC had a minimum one glucose value greater than the 95^th^ percentile, whereas only 12% of the control study population had this finding. HbA1c levels were also found to be 25% higher in the recurrent VVC group when compared to the control group.

Limitations

This study has its limitations because of the limited number of articles published exclusively depicting the direct co-relationship between candidiasis and DM. Several strains of Candida exist, and the studies included in this article do not differentiate amongst which strain is predisposing to more significant susceptibility infections in DM subjects.

## Conclusions

DM is a chronic disease prevalent worldwide, impacting millions of individual's day-to-day lives. The generalized weakness of the immune system and poorly controlled blood glucose levels render the DM patient more susceptible to the development of secondary infections. One such frequently prevalent infection is candidiasis, due to pathogenicity by Candida species due to candidalysin, Sap proteins, and biofilm production. These biofilms resist anti-fungal medications, making the treatment more difficult. Infections of the buccal cavity are widespread in DM patients; oral candidiasis being one of them. Previously conducted research suggests that there are higher chances of developing oral candidiasis in DM subjects than in healthy individuals. Similarly, VVC is also more common in DM patients in comparison to individuals with normal blood glucose levels. Suggestions for future trials include that larger study population groups should be studied. The isolation of Candida strains from people with DM and comparing their counts in healthy subjects is to be done. The impact of periodical screening for VVC on disease outcomes in diabetics should be studied. The role of prophylactic treatment to prevent candidiasis in uncontrolled diabetics can be explored. Further research on this topic would be very helpful in the prompt and effective treatment of patients.

## References

[1] Atsmoni SC, Brener A, Roth Y (2019). Diabetes in the practice of otolaryngology. Diabetes Metab Syndr.

[2] Schmidt AM (2018). Highlighting diabetes mellitus: the epidemic continues. Arterioscler Thromb Vasc Biol.

[3] Rodrigues CF, Rodrigues ME, Henriques M (2019). Candida sp. infections in patients with diabetes mellitus. J Clin Med.

[4] Sopian IL, Shahabudin S, Ahmed MA (2016). Yeast infection and diabetes mellitus among pregnant mother in Malaysia. Malays J Med Sci.

[5] Polke M, Hube B, Jacobsen ID (2015). Candida survival strategies. Adv Appl Microbiol.

[6] Unnikrishnan R, Pradeepa R, Joshi SR, Mohan V (2017). Type 2 diabetes: demystifying the global epidemic. Diabetes.

[7] Ogurtsova K, da Rocha Fernandes JD, Huang Y (2017). IDF Diabetes Atlas: global estimates for the prevalence of diabetes for 2015 and 2040. Diabetes Res Clin Pract.

[8] Guimarães T, Nucci M, Mendonça JS (2012). Epidemiology and predictors of a poor outcome in elderly patients with candidemia. Int J Infect Dis.

[9] Saud B, Bajgain P, Paudel G, Bajracharya D, Adhikari S, Dhungana S, Awasthi MS (2020). Fungal infection among diabetic and nondiabetic individuals in Nepal. Interdiscip Perspect Infect Dis.

[10] Lima A L, Illing T, Schliemann S, Elsener P (2017). Cutaneous manifestations of diabetes mellitus: a review. Am J Clin Dermatol.

[11] Casqueiro J, Casqueiro J, Alves C (2012). Infections in patients with diabetes mellitus: a review of pathogenesis. Indian J Endocrinol Metab.

[12] Gonçalves B, Ferreira C, Alves CT, Henriques M, Azeredo J, Silva S (2016). Vulvovaginal candidiasis: epidemiology, microbiology and risk factors. Crit Rev Microbiol.

[13] Singh S, Fatima Z, Hameed S (2015). Predisposing factors endorsing Candida infections. Infez Med.

[14] Martins N, Ferreira I C, Barros L, Silva S, Henriques M (2014). Candidiasis: predisposing factors, prevention, diagnosis and alternative treatment. Mycopathologia.

[15] Naglik JR, Gaffen SL, Hube B (2019). Candidalysin: discovery and function in Candida albicans infections. Curr Opin Microbiol.

[16] Ho J, Camilli G, Griffiths JS, Richardson JP, Kichik N, Naglik JR (2021). Candida albicans and candidalysin in inflammatory disorders and cancer. Immunology.

[17] Swidergall M, Khalaji M, Solis NV (2019). Candidalysin is required for neutrophil recruitment and virulence during systemic Candida albicans infection. J Infect Dis.

[18] Singh DK, Németh T, Papp A (2019). Functional characterization of secreted aspartyl proteases in Candida parapsilosis. mSphere.

[19] Naglik JR, Challacombe SJ, Hube B (2003). Candida albicans secreted aspartyl proteinases in virulence and pathogenesis. Microbiol Mol Biol Rev.

[20] Donlan RM, Costerton JW (2002). Biofilms: survival mechanisms of clinically relevant microorganisms. Clin Microbiol Rev.

[21] Chandra J, Kuhn DM, Mukherjee PK, Hoyer LL, McCormick T, Ghannoum MA (2001). Biofilm formation by the fungal pathogen Candida albicans: development, architecture, and drug resistance. J Bacteriol.

[22] Rajendran R, Robertson DP, Hodge PJ, Lappin DF, Ramage G (2010). Hydrolytic enzyme production is associated with Candida albicans biofilm formation from patients with type 1 diabetes. Mycopathologia.

[23] Silva S, Rodrigues CF, Araújo D, Rodrigues ME, Henriques M (2017). Candida species biofilms' antifungal resistance. J Fungi (Basel).

[24] Nazir M A, AlGhamdi L, AlKadi M, AlBeajan N, AlRashoudi L, AlHussan M (2018). The burden of diabetes, its oral complications and their prevention and management. Open Access Maced J Med Sci.

[25] Chouhan S, Kallianpur S, Prabhu KT, Tijare M, Kasetty K, Gupta S (2019). Candidal prevalence in diabetics and its species identification. Int J Appl Basic Med Res.

[26] Gunther LS, Martins HP, Gimenes F, de Abreu ALP, Consolaro MEL, Estivalet TIE (2014). Prevalence of Candida albicans and non-albicans isolates from vaginal secretions: comparative evaluation of colonization, vaginal candidiasis and recurrent vaginal candidiasis in diabetic and non-diabetic women. Sao Paulo Med J.

[27] Jhugroo C, Divakar DD, Jhugroo P, Al-Amrid SAS, Alahmari AD, Vijaykumar S, Parine SR (2019). Characterization of oral mucosa lesions and prevalence of yeasts in diabetic patients: a comparative study. Microb Pathog.

[28] Anari AG, Hazar N, Sadrabad MJ, Kharazmi S, Kheirollahi K, Mohiti A, Namiranian N (2019). Comparing the frequency of some oral lesions in prediabetic and healthy individuals: is there any difference?. Int J Prev Med.

[29] Bissong M, Azodo CC, Agbor MA, Nkuo-Akenji T, Fon PN (2015). Oral health status of diabetes mellitus patients in Southwest Cameroon. Odontostomatol Trop.

[30] Goswami R, Dadhwal V, Tejaswi S (2000). Species-specific prevalence of vaginal candidiasis among patients with diabetes mellitus and its relation to their glycaemic status. J Infect.

[31] Mohammadi F, Javaheri MR, Nekoeian S, Dehghan P (2016). Identification of Candida species in the oral cavity of diabetic patients. Curr Med Mycol.

[32] Belazi M, Velegraki A, Fleva A (2005). Candidal overgrowth in diabetic patients: potential predisposing factors. Mycoses.

[33] Zeng X, Zhang Y, Zhang T, Xue Y, Xu H, An R (2018). Risk factors of vulvovaginal candidiasis among women of reproductive age in Xi'an: a cross-sectional study. BioMed Res Int.

[34] Dovnik A, Golle A, Novak D, Arko D, Takač I (2015). Treatment of vulvovaginal candidiasis: a review of the literature. Acta Dermatovenerol.

[35] de Leon EM, Jacober SJ, Sobel JD, Foxman B (2002). Prevalence and risk factors for vaginal Candida colonization in women with type 1 and type 2 diabetes. BMC Infect Dis.

[36] Kendirci M, Koç AN, Kurtoglu S, Keskin M, Kuyucu T (2004). Vulvovaginal candidiasis in children and adolescents with type 1 diabetes mellitus. J Pediatr Endocrinol Metab.

[37] Donders GG, Prenen H, Verbeke G, Reybrouck R (2002). Impaired tolerance for glucose in women with recurrent vaginal candidiasis. Am J Obstet Gynecol.

